# 

**Published:** 1807-11

**Authors:** 


### NEW MEDICAL PUBLICATIONS.

A Letter on the Practice of Midwifery, addressed to Sir James Earle, by John Boys, 1s. 6d.

A Treatise on Hernia, being the Essay which gained the Prise offered by the Royal College of Surgeons, 1806, by William Laurence, 8vo.

Observations on Emphysema, or the Disease which arises from an Effusion of Air into the Cavity of the Thorax, or Subcutaneous Cellular Membrani; by Andrew Halliday. 8vo. 5s. boards.

Additional Cases of Gout, in farther proof of the salutary Efficacy of the Cooling Treatment of that afflicting Disease, with illustrative Annotations, written Authorities in its Support, Controversional Discussions, and a View of the present State and future Prospects of the Practice; by Robert Kinglake. 8vo. 8s. 6d. boards.

### To CORRESPONDENTS.

Mr. Davies, in Answer to Mr. Ring, will appear in our next Number.

The continuation of Dr. Bellamy's Case is received.

